# Understanding the Relationship Between Gender Representation in County Government and Perinatal Outcomes to Black, White, and Hispanic Birthing People in Georgia

**DOI:** 10.1089/whr.2023.0158

**Published:** 2024-03-12

**Authors:** Kaitlyn K. Stanhope, Pragati Kapila, Afsha Hossain, Maha Abu-Salah, Vanshika Singisetti, Amal Umerani, Sierra Carter, Sheree Boulet

**Affiliations:** ^1^ Department of Gynecology and Obstetrics, Emory University School of Medicine, Atlanta, Georgia, USA.; ^2^ Emory College of Arts and Sciences, Atlanta, Georgia, USA.; ^3^ Biology and Women's Studies, University of Georgia, Athens, Georgia, USA.; ^4^ Department of Psychology, Georgia State Uniersity, Atlanta, Georgia, USA.

**Keywords:** local government, maternal and infant health, preterm birth, hypertensive disorders of pregnancy, birthweight

## Abstract

**Objective:**

To characterize the association between percent of county-level elected officials who were female-presenting and perinatal outcomes in Georgia and variation by individual race, 2020–2021.

**Materials and Methods:**

We gathered data on the gender composition of county-level elected officials for all Georgia counties (*n* = 159) in 2022 and calculated the percent of female elected officials (percent female, 0–100). We linked this to data from 2020 to 2021 birth certificates (*n* = 238,795) to identify preterm birth (PTB, <37 weeks), low birthweight (LBW, <2500 grams), hypertensive disorders of pregnancy, and cesarean delivery. We fit multilevel log binomial models with generalized estimating equations, with percent female as the primary independent variable. We adjusted for individual and county-level potential confounders and individual race/ethnicity as an effect modifier.

**Results:**

County median percent female elected officials was 22.2% (interquartile range: 15.5). Overall, 14.6% of births were PTB and 10.1% LBW. A 15 percentage point increase in percent female elected officials was associated with lower risk of hypertensive disorders of pregnancy for white (adjusted risk ratio [RR]: 0.94, 95% confidence interval [CI]: 0.88–0.99), and possibly Hispanic (adjusted RR: 0.95, 95% CI: 0.89–1.0) and non-Hispanic other (adjusted RR: 0.94 (0.87–1.01), but not black birthing people (adjusted RR: 1.0, 95% CI: 0.95–1.05). There was not a clear pattern for PTB, birthweight, or cesarean delivery.

**Conclusion:**

Greater female representation in county government was associated with improved maternal health for some racial/ethnic groups in Georgia.

## Introduction

Women are underrepresented in elected positions at every level in the United States, holding less than a third of seats in the United States Congress, state legislatures, and statewide offices in 2022.^1^ Potentially due to this minority status, female elected officials appear to bring a distinct perspective to politics than their male counterparts, with impacts on agenda-setting and policies.^2^ Once elected to national or state-wide legislative bodies, evidence from the United States and global settings suggest that female legislators may be more likely to propose bills on health, policies to support parental leave, and childcare spending.^2,3^ While there is limited scholarship on local government,^4^ we posit that the same gender dynamics at play in national and statewide executive and legislative bodies are present in county governments and that female elected officials in county governments may support programming on health and families that foster a healthier environment for birthing persons.

An emerging body of work from global context demonstrates improvements in child health following increased gender parity in legislatures.^5^ Evidence from the United States is limited but shows a similar pattern. Homan (2017) showed that greater female representation in U.S. state legislatures was associated with reductions in infant mortality.^6^ At a local level, LaVeist demonstrated improvements in infant mortality among black birthing people in cities with greater representation of black people on city councils.^7^ In California, a higher proportion of county board of supervisor members who were black was associated with increased birthweight and gestational age among black birthing people.^8^ Increasing gender and racial diversity in political representation may impact perinatal health through greater support for social, diversity, and health-related initiatives.^2,5^ Alternately, greater representation in government may represent other characteristics of counties that make it a relatively better place for women.

Prior research comparing across countries has identified correlations between the proportion of female elected officials and a range of indicators of female empowerment, including economic and justice.^9^ Either policies supporting the health and rights women or general female empowerment may result in a healthier environment for preconception and pregnant people, potentially resulting in improved maternal and infant outcomes in places with greater female representation.

Geographic differences in perinatal health outcomes are well documented. Individuals living in rural counties, neighborhoods with greater poverty and segregation, and further from health care experience worse maternal and infant outcomes.^10–13^ While many of these patterns are determined by regional factors, county level governments structure opportunity and access to resources for county residents. Zoning and development policies may either facilitate or discourage the development of affordable housing, integrated neighborhoods, and job growth in the county.^14,15^ Further, counties control law enforcement and fee collection policies and practices, which may impact resident health and quality of life. For example, Davis et al. showed that birthing people residing in counties with a higher reliance on fees and fines for revenue generation had higher odds of low birthweight and preterm birth (PTB).^16^

Perinatal health acts as a barometer of population health. Population health scholars have long used disparities in birth outcomes as indicators of the inequitable distribution of resources across and among populations.^17,18^ Emerging evidence supports the role of social and political context in maternal health outcomes.^11,19^ Pregnancy and birth are critical windows for the infant and birthing person. Prenatal exposures, including social and physical stressors, impact infant development, resulting in short- or long-term vascular, hormonal, and metabolic changes.^20,21^ For the birthing person, pregnancy acts as an acute stressor, possibly causing underlying poor metabolic or vascular health to manifest (*e.g.,* through the development of gestational diabetes or hypertension) and with long-term health implications.^22,23^ By understanding how the political environment relates to health during this window, we can make inference about how exposure to greater or less gender representation in local government impacts health for populations across the life course.

Counties are not homogenous, and some counties may be highly economically and racially polarized. The polices that increase resources and growth in some neighborhoods may, in turn, displace individuals or deprive other neighborhoods of resources.^15,24^ Alternately, policies may provide advantages to some groups of individuals while disadvantaging other groups. For example, school choice policies ostensibly allow students to attend any school in their district. However, for families reliant on public transit, they may be limited in schools that students can access via public transit.^25^ It is possible that gender parity in county government may benefit one group (*e.g*., white women) but the benefits may not extend to other groups within the county. We posit that relatively advantaged communities (*e.g*., white birthing people) may reap greater benefits from increases in female representation compared to relatively disadvantaged communities (*e.g*., birthing people of color).

Extending prior work on the relationship between female representation and perinatal health,^6^ we focus on local representation and include both infant and maternal outcomes. In this study, we focus on two infant outcomes: PTB (<37 weeks gestation) and birth weight (continuous) and two maternal outcomes: hypertensive disorders of pregnancy (binary) and primary cesarean delivery. We selected these outcomes because of their strong association with maternal and infant mortality and morbidity.^26,27^ We consider whether the gender distribution of county-level elected officials is associated with birth outcomes across Georgia's 159 counties. Further, we test whether these associations may differ by maternal race.

## Materials and Methods

### Study population

We used data from the 2020 to 2021 restricted-use Georgia natality files to identify outcomes (PTB [<37 weeks gestation], birthweight [continuous, grams], hypertensive disorders of pregnancy [binary, inclusive of gestational hypertension and eclampsia as recorded on the birth certificate], and primary cesarean delivery [excluding repeat cesarean]). We linked these data to information on the gender distribution of elected officials using county of residence at delivery. This study was approved by the Emory University Institutional Review Board (STUDY00004139).

### Gender composition of elected officials

Study team members collected data on county-level elected officials currently in office in 2022 for all counties in Georgia (*n* = 159). For each county, a trained member of the study team examined the county website and abstracted data for up to twelve currently elected officials. We selected 12 as the cutoff to include all members of the county board of commissioners, sheriff, and tax commissioner for all counties where these positions existed. For counties with few elected officials, we abstracted data on all elected officials. For counties with more than 12 elected officials, we abstracted data on up to 12 elected officials with the following priority order: (1) board of commissioners or supervisors; (2) sheriff; (3) tax commissioner; (4) district attorney; (5) judges or clerks of courts; and (6) other county-wide elected officials (*e.g*., coroner, surveyor).

For each elected official, the study team member collected data on position, term start date, term end date, and, where available, race and gender. For many elected officials, explicit race and gender were not available, and we assigned apparent race and gender based on appearance, and, for gender, name and pronouns used in biographies. We confirmed the elected status of each elected official using a second source (BallotReady, a website including all in office officials and candidates or a county sample ballot). For a 10% sample of all counties, a second data abstractor checked the assigned apparent race and gender to ensure consistency across study team members. Individual level descriptive information on elected officials is available in Appendix Table A1.

We used the information on gender composition of elected officials to create a county-level variable, percent female, indicating the percent of elected officials in office in 2022 who self-identified as or were apparently female. We also created an indicator of the difference between the percent of residents in the county who were female (as measured on the American Community Survey)^28^ and percent of elected officials who were female. This measure could theoretically range from −100 to 100 with greater positive numbers indicating relatively larger proportion of the population who was female compared to the proportion of elected officials who were female. For example, a county where 55% percent of residents were female and 25% of elected officials were female would have a 30-point difference.

### Individual covariates

Differences in the distribution of birthing people across counties may account for all or part of the observed associated between percent female and perinatal outcomes. To account for this, we included information from the birth certificates as individual covariates: maternal race (defined as non-Hispanic white, non-Hispanic black, non-Hispanic Other, or Hispanic), education (defined as less than eighth grade, 9–12 grade, high school graduate, some college or college or higher), maternal age, and insurance status at delivery (uninsured, commercial insurance, public insurance, or other).

### Place-based covariates

We considered that the percent of female elected officials may be intertwined with other elements of the county environment. To characterize counties with higher and lower percent of female-presenting elected officials, we selected county-level characteristics representing domains of social and economic status, inequality (both income and gender based), access to health care, and voter participation. From the 2021 American Community Survey, we included county level percent of adults without a high school education, percent of unemployed adults, percent of households below the federal poverty level, and Gini index.^28^ The Gini index is a measure of county-level income inequality. We also calculate sex-specific measures of unemployment and high school graduation rates and created ratios of the proportion of males to female who were unemployed or who had graduated from high school.

From the March of Dimes, we included information on 2020 designation as a maternity care desert (binary).^29^ From the 2021–2022 Area Health Resource Files,^30^ we include information on the availability of primary and obstetric care providers in the county, presence of one or more Federally Qualified Health Centers, and primary care Health Provider Shortage Area designation. From the Georgia Secretary of State's office, we include information on voter participation (the percent of registered voters who voted) for black and white voters in the 2020 presidential election.^31^ Finally, from the National Center for Health Statistics, we include information on county-level rurality.^32^

### Analysis

For descriptive analysis, we created a binary indicator for having at least 40% of elected officials in the county who were female or not. We selected 40% as the cutoff for descriptive analysis based on the median value of percent female across births to create two equal sized groups for comparison. We present differences in individual characteristics by each maternal race category and, within that, residence in a county with greater than or less than 40% of elected officials who were female. We describe differences in counties with greater than or less than 40% female elected officials.

We fit multivariable models to estimate the association of continuous percent female with each outcome independent of individual or place-based characteristics. For PTB, hypertensive disorders of pregnancy, and cesarean delivery, we fit log binomial models. For birthweight, we fit linear models. We used generalized estimating equations to account for potential clustering by county and calculate a population averaged effect. To select potential confounders, we used *a priori* knowledge and a direct acyclic graph to parsimoniously select a set of county and individual variables that may impact the likelihood of electing female-presenting elected officials and perinatal outcomes. We fit unadjusted and adjusted models for each, adjusting for individual race, individual parity, individual age, individual insurance, and individual education; and county rurality (binary, urban or rural), percent of families below the federal poverty level, and county percent of female residents.

We further considered that the association of percent female with each outcome may vary by maternal race/ethnicity and included an interaction term between maternal race/ethnicity and percent female in all models. We estimated differences in outcomes with a 1-standard-deviation (SD) (15 percentage point) difference in the percent of female elected officials.

## Results

We included 229,802 singleton births to Georgia residents in 2020–2021. Overall, 43.3% (99,616) were to white birthing people, 34.2% (78,678) to black birthing people, 15.5% to Hispanic birthing people, and 6.9% to birthing people who identified as another racial or ethnic background (Table 1). In counties with a lower proportion of female-presenting elected officials, birthing people were more likely to be adolescents, more likely to report Medicaid insurance for delivery, and less likely to have a college degree, within all race categories. A slightly higher proportion of birthing people in counties with a higher percent of female-presenting elected officials entered care in the first trimester within all race categories. In unadjusted comparison, the risk of infant complications (PTB, low birthweight) was lower within all race groups in counties with a higher percent of female-presenting elected officials. For maternal outcomes, this held for gestational hypertension and eclampsia but not cesarean delivery.

**Table 1.** Individual Characteristics of Georgia Births by Gender Composition of County Elected Officials, Stratified by Maternal Race/Ethnicity, *n* = 229,802

***N*** births	White		Black		Non-Hispanic Other		Hispanic	
	<40% Female	40%+ Female	<40% Female	40%+ Female	<40% Female	40%+ Female	<40% Female	40%+ Female
	62,015	37,601	31,153	47,525	5,337	10,534	12,993	22,644
	% (***n***)	% (***n***)	% (***n***)	% (***n***)	% (***n***)	% (***n***)	% (***n***)	% (***n***)
Age (mean, SD)	28 (8)	31 (7)	26 (9)	29 (9)	30 (8)	31 (7)	27 (9)	28 (10)
Age category								
>20	8.2 (5,056)	3.9 (1,454)	13.8 (4,306)	8.4 (4,011)	8.7 (462)	3.2 (341)	12.6 (1,637)	11.3 (2,568)
20–34	78.9 (48,903)	73.6 (27,669)	74.3 (23,149)	72.3 (34,346)	72 (3,843)	71.3 (7,507)	70.9 (9,209)	69.4 (15,716)
35+	13 (8,056)	22.6 (8,478)	11.9 (3,698)	19.3 (9,168)	19.3 (1,032)	25.5 (2,686)	16.5 (2,147)	19.3 (4,360)
Insurance^a^								
Uninsured	2.4 (1,495)	3.2 (1,184)	1.2 (370)	3.1 (1,487)	4.3 (231)	3.6 (379)	28.6 (3,717)	22.5 (5,102)
Medicaid/public	48.2 (29,891)	28.5 (10,731)	77.8 (24,235)	62.9 (29,885)	47.6 (2,538)	33.1 (3,481)	49.1 (6,374)	50.1 (11,333)
Commercial	47.8 (29,613)	67.6 (25,408)	19.4 (6,050)	32.7 (15,515)	46.2 (2,466)	62.7 (6,604)	20.6 (2,671)	24.2 (5,472)
Other	1.6 (1,016)	0.7 (278)	1.6 (498)	1.3 (638)	1.9 (102)	0.7 (70)	1.8 (231)	3.3 (737)
Married	68.4 (42,443)	78.4 (29,458)	23.4 (7,280)	32.8 (15,589)	67.3 (3,589)	79.1 (8,328)	50.9 (6,607)	47.6 (10,780)
Education								
0–8 grade	1.1 (651)	0.8 (291)	0.8 (239)	1 (469)	1.4 (76)	2.7 (279)	16.7 (2,163)	12.8 (2,886)
9–11 grade	8.5 (5,287)	4.6 (1,731)	11.3 (3,524)	7 (3,329)	7.6 (408)	4.6 (486)	15.2 (1,972)	12.9 (2,923)
High school	29.8 (18,451)	18.9 (7,108)	45 (14,013)	35.6 (16,916)	26.3 (1,404)	18.8 (1,982)	36.2 (4,701)	44 (9,954)
Greater than high school,	29.4 (18,239)	19.3 (7,240)	29.9 (9,304)	27.5 (13,087)	23.8 (1,271)	16.1 (1,691)	19.4 (2,520)	14.4 (3,254)
College, or more	31.3 (19,387)	56.5 (21,231)	13.1 (4,073)	28.9 (13,724)	40.8 (2,178)	57.9 (6,096)	12.6 (1,637)	16 (3,627)
Pregnancy characteristics								
Number of live births (Including current)	2 (2)	2 (2)	2 (3)	2 (2)	2 (2)	2 (2)	2 (2)	2 (2)
Entered prenatal care in first trimester	81.5 (50,509)	86.4 (32,488)	71.5 (22,275)	70.9 (33,683)	76.3 (4,072)	79.1 (8,332)	63.1 (8,202)	71.1 (16,109)
Infant outcomes								
PTB	10.5 (6,521)	10.3 (3,853)	17 (5,308)	15.7 (7,473)	10.8 (578)	12.9 (1,363)	12.1 (1,573)	14.4 (3,249)
Very PTB	1.2 (770)	1.1 (415)	3 (931)	2.7 (1,266)	1.6 (84)	1.8 (188)	1.4 (183)	1.8 (407)
Low birth weight (<2,500 g)	6 (3,694)	5.3 (1,997)	13.1 (4,084)	12.1 (5,764)	8.2 (436)	8.3 (879)	6.3 (814)	6.4 (1,439)
Very-low birthweight (<1,500 g)	0.8 (499)	0.7 (256)	2.4 (735)	2.1 (1,003)	1.1 (60)	1 (105)	0.9 (111)	0.9 (211)
Birthweight, grams	3,355 (637)	3,374 (625)	3,095 (632)	3,130 (650)	3,230 (620)	3,190 (620)	3,295 (625)	3,280 (622)
Maternal outcomes								
Gestational Hypertension	10.3 (6,371)	7.6 (2,843)	10.3 (3,210)	9.2 (4,388)	6.5 (345)	4.1 (432)	6.3 (822)	4.2 (953)
Eclampsia	0.2 (118)	0.1 (54)	0.3 (80)	0.2 (93)	0.1 (6)	0.2 (16)	0.1 (17)	0.1 (27)
Primary Cesarean^b^	23.3 (12,334)	24.6 (8,210)	25.4 (6,552)	26.9 (11,200)	23 (1,040)	25.6 (2,384)	18.1 (2,013)	20.8 (4,171)

^a^ Missing for 239 observations.

^b^ Denominator excludes repeat cesarean deliveries.

PTB, preterm birth; SD, standard-deviation.

**FIG. 1.**
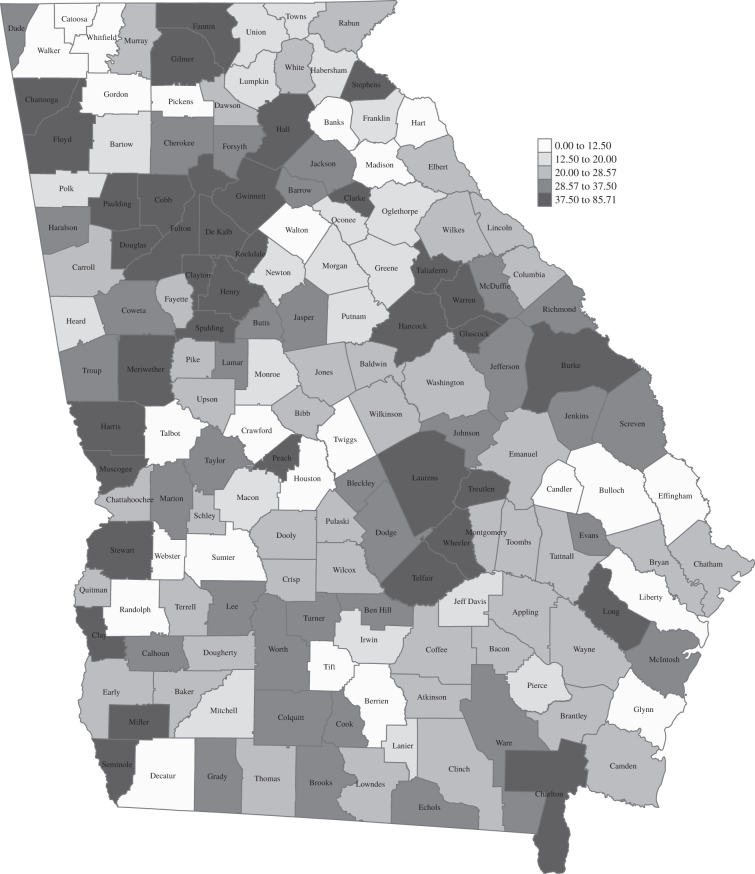
Distribution of percent of county-level elected officials in office in 2022 with a female-presenting gender.

Across the 159 counties in Georgia, the median percent of elected officials who were female-presenting was 22.2 (interquartile range [IQR]: 19) (Figure 1, Table 2). This value ranged from 0 to 85.7%. Only in 31 counties were greater than 40% of elected officials female-presenting. Individual information on elected officials for whom data were abstracted is available in Appendix Table A1. Counties with greater than 40% of female-presenting elected officials had a higher percent of nonwhite elected officials, higher population size, higher black voter participation, and lower percent that qualified as a Health Care Provider Shortage Area for primary care.

**Table 2.** County Characteristics by Gender Composition of County Elected Officials, *n* = 159 Counties in Georgia

***N*** counties	Total	Less than 40% female elected officials	Greater than or equal to 40% female elected officials
	159	128	31
	Median (IQR)	Median (IQR)	Median (IQR)
Count elected officials	9 (2)	9 (2)	9 (3)
Percent of nonwhite elected officials	11.1 (22.2)	10 (20)	28.6 (48.9)
Percent of female elected officials	22.2 (19)	20 (16.1)	42.9 (10)
County population size	23,211 (46,111)	21,798.5 (32,211.5)	27,822 (194,135)
Percent of female residents	50.9 (2)	50.7 (1.8)	51.4 (1.7)
Representation difference	26 (19.1)	29.1 (14.5)	7.2 (18.4)
Median Household income	49,325 (18,279)	48,751.5 (15,898.5)	51,945 (24,760)
Percent of households below federal poverty level	18.2 (9.6)	18.2 (9.1)	17.4 (10.9)
Total population with less than a high school diploma	16.2 (7.4)	16.5 (7.4)	15 (8.8)
Ratio of male to female high school graduates	1.1 (0.2)	1.1 (0.2)	1.2 (0.2)
Ratio of male to female unemployment	0.8 (1.5)	0.8 (1.7)	0.8 (0.9)
Gini Index	0.5 (0.1)	0.5 (0.1)	0.5 (0.1)
Percent of households with females listed as heads of households	15 (5.5)	14.8 (5.2)	16.2 (6.5)
Dissimilarity index	0.4 (0.2)	0.4 (0.2)	0.3 (0.2)
White female voter participation^a^	73.1 (5.4)	73.2 (5.6)	72.6 (4.5)
White male voter participation^a^	72 (5.8)	72.1 (5.8)	72 (5.7)
Black female voter participation^a^	73.1 (5.4)	65.1 (9.9)	67.1 (8.8)
Black male voter participation^a^	52 (10.7)	51.8 (10.1)	53.3 (10.6)
Number of primary care providers	9 (22)	8.5 (17)	12 (112)
Maternity Care Desert (binary)	35.2 (56)	35.9 (46)	32.3 (10)
Number of OB/GYN providers	1 (4)	1 (3)	2 (16)
Full primary care HPSA	62.3 (99)	65.6 (84)	48.4 (15)
Rurality designation, % (*n*)			
Large central metro	0.6 (1)	0	3.2 (1)
Large fringe metro	17.6 (28)	14.1 (18)	32.3 (10)
Medium metro	9.4 (15)	9.4 (12)	9.7 (3)
Small metro	18.9 (30)	20.3 (26)	12.9 (4)
Micropolitan	17.6 (28)	21.1 (27)	3.2 (1)
Noncore	35.9 (57)	35.2 (45)	38.7 (12)

^a^ Defined as the percent of registered voters who voted in the most recent (2020) November election.

HPSA, Health Provider Shortage Area; IQR, interquartile range.

However, median household income, poverty rates, high school graduation rate, inequality (Gini Index), and percent of counties classified as a maternity care desert were similar. A higher percent of counties with low (<40%) percent female were medium and large fringe metro counties (suburban); whereas a higher percent of counties with high (> = 40% female) were micropolitan (small towns). We also calculated the difference between the percent of female residents and percent of female elected officials. The difference ranged from −35.1 (a greater percent of female elected officials relative to the population) to 34.71 (a greater percent of residents were female relative to elected officials) with a median of 26.0 (IQR: 18.5).

In adjusted and unadjusted models, a 1-SD increase in the proportion of female-presenting elected officials was not associated with improved infant outcomes (PTB and birthweight) for white or black birthing people (Table 3). For non-Hispanic other and Hispanic birthing people, the risk of PTB increased and mean birthweight decreased with increasing percent of female-presenting elected officials. For maternal outcomes, there was a lower risk of hypertensive disorders of pregnancy with increasing percent of female elected officials for white, non-Hispanic other, and Hispanic birthing people but not black birthing people. There was no apparent difference for primary cesarean delivery risk.

**Table 3.** Models Estimating Association Between a 1-Standard Deviation (15 Percentage Point) Increase in Percent Female of County Elected Officials in Office in 2022 and Perinatal Outcomes, Georgia 229,802 Births in 2020–2021

	PTB	Birthweight	Hypertensive disorders of pregnancy	Primary cesarean
	RR (95% CI)	Beta (95% CI)	RR (95% CI)	RR (95% CI)
Unadjusted				
Non-Hispanic white	0.98 (0.96 to 1.01)	0.5 (−7 to 8.1)	0.96 (0.91 to 1.01)	1.01 (0.98 to 1.04)
Non-Hispanic black	0.98 (0.95 to 1)	8.2 (−0.7 to 17.1)	1.01 (0.97 to 1.06)	1.02 (1 to 1.04)
Non-Hispanic other	1.01 (0.96 to 1.07)	–15.4 (−33.3 to 2.5)	0.97 (0.9 to 1.05)	1.02 (0.99 to 1.05)
Hispanic	1.02 (0.98 to 1.05)	–12.7 (−22.4 to −2.9)	0.99 (0.93 to 1.05)	1.02 (0.99 to 1.05)
Adjusted^a^				
Non-Hispanic white	0.99 (0.97 to 1.01)	–2.7 (−7.6 to 2.2)	0.94 (0.88 to 0.99)	0.98 (0.95 to 1)
Non-Hispanic black	0.99 (0.98 to 1.01)	4.2 (−1.3 to 9.7)	1.00 (0.95 to 1.05)	0.99 (0.97 to 1.01)
Non-Hispanic other	1.03 (0.98 to 1.07)	–18.7 (−34.2 to −3.1)	0.94 (0.87 to 1.01)	0.98 (0.96 to 1.01)
Hispanic	1.00 (0.96 to 1.04)	–7.9 (−15.5 to −0.3)	0.95 (0.89 to 1)	1 (0.97 to 1.03)

^a^ Adjusting for race, parity, age, insurance, maternal education, rurality, percent of families below the federal poverty level, and percent of female residents.

CI, confidence interval; RR, risk ratio.

## Discussion

In this analysis of elected officials in office in Georgia in 2022, greater female representation in local government was associated with improved maternal but not infant outcomes. This provides only limited support for our hypothesis that greater female representation in county government would improve outcomes for birthing people and infants. We identified differences in the direction and magnitude of associations by race. For only white birthing people, a greater overall percent of female-presenting elected officials was associated with reduced risk of hypertensive disorders of pregnancy.

To our knowledge, there are no prior analyses of the distribution of gender among county-level elected officials and perinatal outcomes. However, there is a large body of work globally on how female political participation and representation can improve infant outcomes and some research on state-level gender variation in the United States.^2,6^ Prior researchers have found reduced infant mortality in places with greater female representation, including across U.S. states.^6,33^ In contrast, we did not find meaningful reductions in preterm or low-birth-weight birth for most birthing people in Georgia with greater female representation.

Further, prior researchers have considered how racial representation in county and city governments impacts infant outcomes, identifying reduced infant mortality and reduced PTB risk in places with a greater proportion of black representatives in local government.^7,8,34^ The lack of an association between a greater proportion of female-presenting people in county governments and infant outcomes in our study may be due to our incorporation of effect modification by individual race, the distinct context of Georgia, or the relative obscurity of county elected officials (compared to state or city-wide officials, who may have greater name/face recognition for residents).

This cross-sectional analysis precludes an assessment of causality; there are at least two distinct scenarios that may have generated the observed association between female representation and lower risk of hypertensive disorders. First, counties with greater gender equity overall, particularly for white women, may elect more female elected officials. The more equal ratios of male-female high school graduates and male-female unemployment shown in Table 2 provide some support for this. Second, female elected officials may bring a different perspective to their role, intentionally or implicitly choosing policies that better support the health of women. Historic evidence at the national level supports this idea.^2,3^ In our data, we observed greater numbers of primary care providers in counties with at least 40% female elected officials compared to less than 40% female elected officials.

Future researchers may consider analyses of the political action of local female elected officials and the contexts that give rise to them to better understand which pathways may be at work.

Whether due to policy actions or empowerment, the potential benefits of more gender equity or greater female representation did not appear to extend to black birthing people in this sample. We speculate that this may be due to two primary reasons. First, primarily white female elected officials likely do not represent the unique perspectives of black women. Evidence from political science shows that black women bring a unique political perspective to elected office, with different agendas than their male counterparts or white females.^35^ Second, black women and other black birthing people enter pregnancy with a lifetime of exposure to overlapping systems of oppression, including national and state-level political contexts with few black women in elected office.^17,36,37^ The potential positive impact of female representation may be blunted within this historic and continued adverse context.

### Implications for policy

Political scientists have identified a number of barriers to women serving in political office. In an analysis of graduates of a candidate prep workshop, Bernhard et al. found that women who were the primary breadwinner for their families were less likely to run for office at any level.^38^ The majority of elected officials identified by our team were white men. Further, the majority of female elected officials were white. Race and gender may interact to form greater barriers for women of color in running for and being elected to public office, regardless of qualifications.^39^ This may, in part, explain why the presence of greater female elected officials in the resident county was not associated with reduced risk of hypertensive disorders of pregnancy for black birthing people.

Future researchers may consider further analyses of the ways in which residents interact with their county government and specific county level policies that may promote or act as barriers to optimal perinatal health—and for whom. For example, researchers in Georgia have identified wide variation in how evictions are handled across counties with similar demographic and social characteristics with critical implications for renting families.^40^ Further, researchers may consider other correlates of places with greater numbers of female-presenting people in public office, including factors that predisposes places to elect female-representing officials and factors that influence female-presenting people's decisions to run for office. Finally, future researchers may consider how political affiliation intersects with gender in promoting health.

### Limitations

The results of this analysis should be considered in light of several limitations. First, the data on elected officials and births are cross-sectional and the birth data predate the data on elected officials. However, we note that, where available, many elected officials had been in office for a median of 3 years (with a wide range; Appendix Table A1). Elected officials who were not recently elected were more likely to lack a starting date for their position, and the true median is likely longer. Second, gender was assigned by a researcher in most cases, and we do not know if that would align with self-reported gender. Third, data on birth outcomes were drawn from the birth certificate. While birthweight and gestational age are known to be accurate (positive predictive value of PTB ∼98%),^41^ data on cesarean delivery and hypertensive disorders of pregnancy are of moderate accuracy.^42^ Further, the birth certificate does not explicitly ask about preeclampsia, precluding an analysis of the distinct hypertensive disorders.

## Conclusions

County governments shape the physical and social environments in which people live before, during, and following their pregnancies. These exploratory results suggest that representation in county government may impact maternal health and there may be differential associations by race/ethnicity. Further research into how individuals interact with their county government and how changes in representation over time impact perinatal health may further elucidate this relationship. These results add to the body of work showing the relationship between political environment and health.

## Abbreviations Used

CI: confidence interval
HPSA: Health Provider Shortage Area
IQR: interquartile range
PTB: preterm birth
RR: risk ratio
SD: standard-deviation

## Appendix

**Appendix Table A1.** Characteristics of included county-level elected officials in office in Georgia in 2022 by presenting-gender, *n* = 1,294^a^

	Female	Male
N	351	935
	% (*N*)	% (*N*)
Race		
White	72.6 (247)	82.2 (731)
Black or African American	26.8 (91)	17.1 (152)
Asian		4.0 (0.45)
Not available	0.6 (2)	0.2 (2)
Position		
Member, county board of supervisors or commissioners	27.1 (95)	51.0 (477)
Sheriff	1.1 (4)	16.4 (153)
Tax commissioner	31.1 (109)	4.7 (44)
Clerk of court	26.2 (92)	2.1 (20)
Solicitor general	1.4 (5)	1.8 (17)
Chair, county board of supervisors or commissioners	5.1 (18)	12.5 (2)
County attorney	0 (0)	0.5 (115)
Other	7.1 (25)	8.9 (83)
District attorney	0.9 (3)	2 (12)
Explicit race available?	5.2 (18)	6.4 (59)
Explicit gender available?	67 (235)	74.3 (695)
Year of Entry into position (median [range])^b^	2019 (1972–2022)	2019 (1972–2022)

^a^ Excluding 75 elected officials without gender information available.

^b^ Missing for 790 elected officials.
